# Will clinical signs become myth? Developing structured Signs Circuits to improve medical students’ exposure to and confidence examining clinical signs

**DOI:** 10.1080/10872981.2022.2050064

**Published:** 2022-04-07

**Authors:** Dominic Merriott, George Ransley, Shadman Aziz, Krushna Patel, Molly Rhodes, Deborah Abraham, Katba Imansouren, Daniel Turton

**Affiliations:** a Intensive Care Registrar, Austin Health, Melbourne, VIC, Australia; b Internal Medicine Trainee, University College London Hospitals NHS Foundation Trust, London, UK; c Emergency Medicine Specialty Trainee, London, UK; d Foundation Doctor, King’s College Hospital NHS Foundation Trust, London, UK; e Foundation Doctor, Barts Health NHS Trust, London, UK; f Anaesthetist at Barts Health and Honorary Lecturer at Queen Mary’s University of London, UK

**Keywords:** Physical examination, clinical signs, examination skills decline, clinical skills

## Abstract

**Background:**

Correctly eliciting and interpreting physical examination (PEx) signs contributes to successful diagnosis and is fundamental to patient care. A significant decline in the time spent acquiring these skills by medical students, and the decreased ability to elicit and recognise signs is widely acknowledged. However, organising teaching to counteract this in the busy clinical environment is challenging. We evaluated the prior exposure to clinical signs, and experience of examination teaching among a cohort of final-year medical students. Following this, we assessed the utility of a structured circuit-based approach (Signs Circuits) using hospital inpatients and junior doctors to provide high-yield PEx teaching and overcome these limitations.

**Materials and methods:**

Qualitative and quantitative survey feedback, including a standardised list of 62 clinical signs, was sought from final-year medical students during their rotations at a teaching hospital in London, UK, before and after the provision of Signs Circuits.

**Results:**

Prior to the course the 63 students reported limited exposure to even the most common clinical signs. For example, the murmurs of mitral and tricuspid regurgitation and the sound of lung crackles eluded 43%, 87%, and 32%, respectively. From qualitative feedback, the reasons for this included that much of their prior PEx experience had focused on the performance of appropriate examination steps and techniques in patients without pathology. During the course, students were exposed to an average of 4.4 new signs, and left with increased confidence examining and eliciting signs, and a firmer belief in their importance to diagnosis.

**Conclusion:**

Medical students continue to have limited exposure to clinical signs in medical school. This signs-focused approach to PEx teaching is an effective and reproducible way to counter the deficiencies identified in signsexposure.

## Background

Physical examination (PEx) skills are fundamental to the process of clinical assessment and diagnosis. Over 25% of patients may have significant clinical signs, which if recognised can change management [1]. Mistaken or missed clinical findings contribute towards medical errors and adverse events [2], with diagnostic errors among even the most senior doctors surprisingly high [3]. Therefore, to make effective doctors, medical students must be exposed to numerous physical signs and feel confident eliciting them and identifying their significance.

However, numerous reports describe the decline of bedside teaching in modern medical practice [4,5], a contributor to declining examination skills. Studies of teaching programmes have shown a shift from bedside to computer-side [6,7]. In dedicated teaching rounds for students by senior physicians in North America, as few as 17% were focused on bedside teaching [8] with some students and trainees finding 75% of teaching rounds avoid the bedside altogether [9].

This is of concern, because an inability to tie clinical and laboratory findings together is a common source of diagnostic errors [10,11], and a leading cause of healthcare litigation [11]. While the increased digitalisation of the healthcare environment offers many benefits, the increased utilisation of this space in teaching risks worsening these trends, putting future patients at risk of diagnostic errors and harm.

While students and trainees believe bedside teaching is the most effective way of learning clinical skills [12], around half report not receiving enough during their training [12,13] with much of their time being spent on the wards unsupervised performing routine, low-acuity tasks [14]. Some suggest that this limited instruction leaves students accustomed to performing a rote organ-system examination but lacking the skills to recognise the signs they encounter and synthesise the information gathered in clinical practice into a diagnosis [5,15]. Expecting these skills to be fully developed as junior doctors is unwise, with studies showing only a small fraction of time remains for direct patient care outside of their administrative duties [16] and that some examination skills may not improve after latter years of medical school and into physician training [17].

The decline of bedside teaching, although acknowledged, is difficult to remedy due to multiple factors [18,19]. These include real or perceived difficulties recruiting patients; allocating teaching time to busy doctors, particularly senior clinicians who have large pre-existing commitments; decline of practitioner teaching skills [20]; session delivery in the busy clinical environment [8]; and lack of consensus over the most effective strategies for improving PEx skills [21,22]. Most recently, concerns over infection control in the era of COVID-19 have put a halt to many bedside programmes.

In this context, we hypothesised that students in a teaching hospital in London (UK) would lack exposure to many relevant clinical signs. This study aims to evaluate the degree of this exposure gap and assess the impact of a structured approach to bedside teaching on final-year student’s clinical signs exposure and confidence in PEx skills.

## Methods

### Innovation

‘Signs Circuits’ is a novel teaching programme established at four hospital sites across London (UK) with the aim of improving medical students’ exposure to clinical signs. The course seeks to provide exposure to hospital inpatients who have diagnostic clinical signs with direct supervision and feedback from junior doctor tutors. A circuit structure, with small groups of students moving between each patient-and-tutor ‘station’, was chosen. Patients are seen in their hospital beds to minimise disruption, with a tutor staying with them throughout the circuit as student groups travel between beds or wards as necessary. The philosophy is centred around high-yield sessions that over a short period of time (1.5–2 hours) can provide focused teaching on four patients with relevant clinical signs. This makes for a time efficient teaching session that fits in with student and tutor schedules.

Patients are recruited by course leads in the days prior to the teaching sessions. Teaching is performed predominantly by a separate cohort of postgraduate year 1–2 junior doctors who are given time to familiarise themselves with the clinical sign(s) and devise an approach to the station. Informed consent from the patients is gained in advance, and in case of patient withdrawal, backup patients are recruited, and computer-based stations are devised as a fall-back.

In the iteration presented here, from between 2019 and 2020, weekly circuits of three to four inpatients were arranged for the duration of final-year students’ 6-week rotations. To keep group sizes small (2–4 students), the cohort was split into two groups who attended alternate weeks, providing each student with three circuits. Each patient station lasted 25 minutes, with time for each student to perform a general inspection and focused examination, followed by feedback from the tutor on examination skills, the sign(s), and its clinical relevance. Students were encouraged to re-examine to consolidate knowledge before rotating around the circuit.

### Study design

We conducted a repeated measures longitudinal cohort study of final-year medical students before and after participation in Signs Circuits. This study received ethical approval and was registered as QMREC2360a with Queen Mary University of London (QMUL).

### Study population

All final-year medical students rotating through our hospital taking part in Signs Circuits were asked to provide quantitative and qualitative feedback. All provided consent to use their anonymous survey results in further research.

### Data collection

To assess prior bedside teaching and the impact of Signs Circuits, pre- and post-course questionnaires were designed (Appendix 1) with responses given as white-space written answers, or self-graded Likert Scale responses (1 – Strongly Disagree to 5 – Strongly Agree). In addition, a standardised list of 62 clinical signs were compiled from clinical textbooks and discussion with teaching faculty. Students were asked to record whether they had been exposed to these signs in their training to date, and post-course whether they had now observed it.

### Data analysis

Questionnaire data was analysed using Microsoft Excel. Mean Likert scores were calculated, and two tailed *T*-tests performed to deduce significance of the difference between means. The alpha value was set at 0.05.

## Results

Sixty-three students were taught as part of Signs Circuits over a 6-month period in four cohorts. All completed a pre-course questionnaire, and 31 the post-course questionnaire. Due to the COVID-19 pandemic, the fourth cohort of students started but were unable to complete the course or provide post-course feedback. Data from this group was included for analysis of students’ previous experience of clinical teaching but excluded from comparative measures of responses before and after completing the course. Most post-course survey respondents attended two Signs Circuits teaching sessions (19/31, mean number of sessions 2.32).

The main outcome measures (Table 1) showed significant improvement pre- to post-course, with students’ self-reported measures of their comfort examining patients and eliciting signs improving. Students also reported a stronger conviction that clinical signs were important to diagnosis.

**Table 1.** Pre- and post-course quantitative feedback regarding physical examiatnion

	Pre-course mean Likert Score [^1–5^]	Post-course mean Likert Score [^1–5^]	*T*-Stat	*p*-Value	95% Confidence interval for difference of means
I feel very comfortable examining patients	3.52	3.87	2.45	0.01	0.07–0.63
I feel very comfortable consistently eliciting clinical signs	2.84	3.68	5.62	<0.01	0.54–1.13
Clinical signs are a pivotal part of diagnosis	4.33	4.71	2.30	0.02	0.05–0.70

### Exposure to clinical signs

Full data for the clinical signs seen pre-course is listed in Appendix 2. Pre-course, the average total number of signs seen per student was 30.4; post-course, this increased to 36.6. However, the difference between the means was not significant (p = 0.1, CI −1.40 to 13.79, post-hoc power analysis 32.1%). The average number of new signs students had encountered during their placement was 4.39 (SD 1.92, range 0–38).

Key signs are summarised in Table 2, showing that many common signs, important to diagnosis and management, had never been observed by a substantial proportion of participants.

**Table 2.** Pre-course exposure to select clinical signs

Clinical signs	Observed pre-course (% total)
**Cardiovascular**	
Aortic stenosis	81%
Aortic regurgitation	43%
Mitral regurgitation	57%
Mitral stenosis	32%
Tricuspid regurgitation	13%
Raised JVP	59%
S3 heart sound	16%
S4 heart sound	10%
**Respiratory**	
Crepitations	68%
Bronchial breathing	50%
Deviated trachea	6%
**Abdominal**	
Hepatomegaly	50%
Splenomegaly	27%
Spider naevi	51%
Scleric icterus	24%
**Neurology**	
Hypertonia	78%
Hypotonia	56%
Hyperreflexia	52%
Babinski’s sign	27%

### Feedback on Signs Circuits

Direct feedback on Signs Circuits including quality, interactivity, and the level at which it was aimed was almost universally positive, with 99% of students agreeing or strongly agreeing to these questions.

Post-course qualitative feedback was strongly positive. Asked what made this type of teaching good, students emphasised the teaching quality, interactivity, good organisation, and exposure to numerous clinical signs. Some students also drew attention to the provision of feedback: ‘A chance to get feedback and learn whether what you heard was actually what you thought you heard’.

Asked what had been most useful to see, the most common answers were cardiac signs including murmurs, and respiratory signs. This was repeated in the signs they would like to have seen more of, with others requesting more neurology signs.

Asked what improvements could be made, 21/31 students (68%) responded that they would like more sessions. Others suggested more time with each patient, and more patients per circuit.

Of the 31 students, 22 (71%) responded that the course would improve their confidence, knowledge or consistency examining patients, or would be useful for their upcoming OSCEs. Three mentioned planning to set up similar teaching programs when they started working, and one responded: ‘It was a very good idea and would be a great part of every hospital rotation’.

### Previous PEx teaching

**Figure 1.**
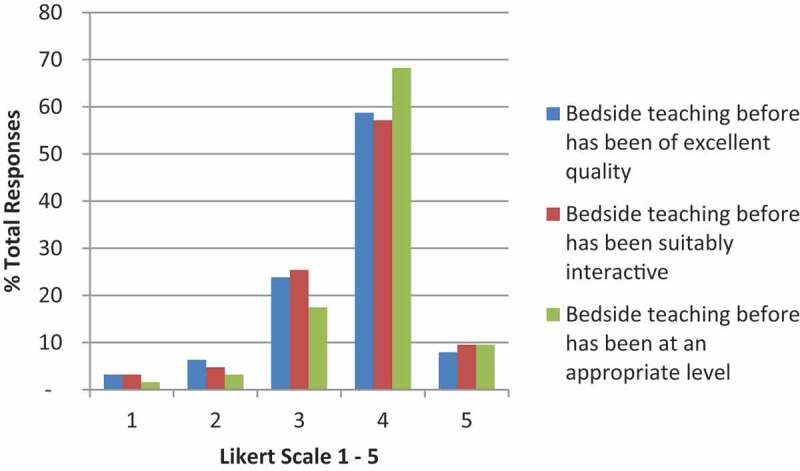
Students experience of previous bedside teaching. Self-graded by likert scale 1–5 (strongly disagree to strongly agree).

Students reported receiving limited prior PEx teaching (mean Likert score 3.02), and pre-course 97% were unsure, or disagreed with the statement that they felt prepared for their final OSCEs (mean Likert score 2.37) (Figure 1).

Asked whether they were shown signs by doctors during placements, most responded yes or sometimes. Many observed that it was mainly FY1s (first-year postgraduates), or other juniors doing this. Many students though echoed the sentiments of one student who replied: ‘ … sometimes they point us in the right direction to relevant patients, but rarely [are we] observed’. Some highlighted that when taught, examination technique was the focus with one student commenting on being‘well-practiced at examining “normal” … but not “abnormal”’.

**Figure 2.**
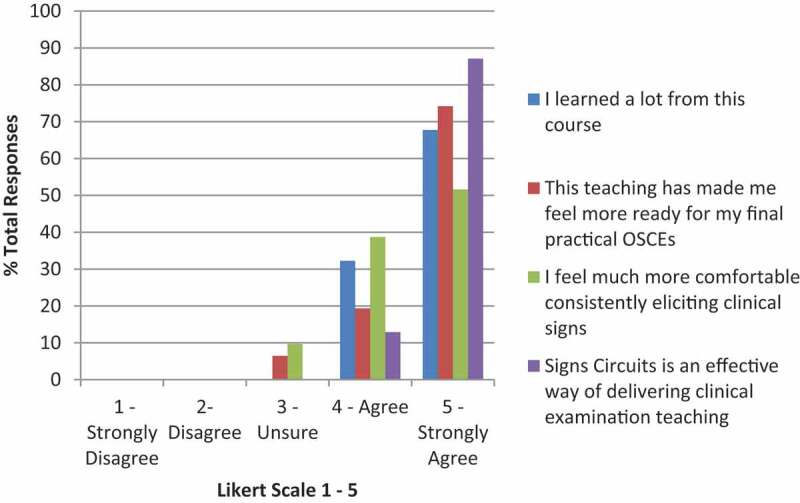
Post-course self-reporting on the impact of the Signs Circuits on students. Self-graded by likert scale 1–5 (strongly disagree to strongly agree).

Pre-course, reflecting on what factors made previous teaching good, the majority highlighted smaller groups, and more interactivity between student and teacher. Poor teaching was characterised by larger groups, rushed delivery, and feeling put on the spot or grilled by the tutor (Figure 2).

## Discussion

These results identify deficiencies in medical student clinical examination teaching and weaknesses recognising common clinical signs. The response from students affirms the value of focusing a teaching strategy on increasing exposure to clinical signs.

### Student experience in context

Students could not recall personally examining numerous signs common among hospital inpatients. While unable to find similar data, numerous studies have reported deficiencies in students and trainees accurately identifying clinical signs [^23–25^]. Our research accords well with this, as the ability to identify a sign in practice is likely to be limited if not exposed to it in training. In clinical practice this could have significant implications involving over or under-investigating for numerous diseases where examination findings can be discriminatory.

Our students seemed to understand their limitations and lack of experience. Many expressed frustration at their limited PEx teaching and similar to other studies much of their examination practice came unsupervised [26], often having merely been directed towards a patient by busy junior doctors. Accordingly, many lack the confidence to examine patients independently, unsure of the purpose of attempting to elicit signs when they are unsure of their interpretation.

### Strategies for bedside teaching

Many PEx teaching programmes report on successes in important but narrow outcomes such as the ability to perform a specific musculoskeletal system examination [^27–29^]. Whilst there are other innovative approaches aimed at delivering focused examination teaching that can be done on an individual level [30,31], there is little evidence on how to implement structured bedside teaching that focuses on the multi-system diagnostic skills required of junior doctors.

Further, all teaching is done in the context of the OSCE examination. For our students, this means only needing to perform a competent, systems-based examination and generating a sensible differential diagnosis to pass. There is much literature discussing whether this approach encourages rote learning of a run-through exam, without the ability to apply those skills to clinical dilemmas to reach a diagnosis. Notably, OSCE scores may not correlate with focused tests of students’ diagnostic skills [5,32], with these skills better reflected by the amount of time senior physicians have spent with students reviewing exam findings at the bedside [32]. Some advocate the development of the ‘hypothesis-driven physical examination’ to counteract this [15], creating a more signs and diagnosis-focused examination style.

This informed our philosophy to structure sessions around a focused examination, often concentrating on just one or two signs, followed by feedback, and re-examination. This may mean an entire station spent listening and re-listening to heart sounds. This avoids information overload, well known to be associated with worse retention [33]; and gives ample time to focus on direct task-specific feedback, one of the most important aspects of good teaching [34,35], and one that our students, and many studies, reported is often lacking [36].

### Challenges in providing bedside teaching

Previously in our institutions, bedside teaching largely relied on a single teaching fellow directing small group teaching, or informal junior doctor mentors who may lack either the support to provide consistent structured teaching, or the time to find patients and provide teaching that went beyond an OSCE-based examination. In these structures, the Signs Circuits approach, with designated leads and teaching support from a large cohort of junior doctors, presented a formalised, but labour-intensive change. For the leads, this meant finding time to consent up to 6 patients per week, and for the tutors, being available for 2 hours of teaching during the workday. To ensure teaching is high quality and accurate, tutors should be supervised by teaching fellows, and should only teach established diagnoses, guided by senior opinion and investigation findings.

Other challenges are also apparent. The circuit could be tiring for patients and interfere with clinical activities. Patients must therefore be aware that they can withdraw at any time, and leads must liaise with ward staff to ensure only appropriate patients are recruited.

Timing of sessions is a further consideration. In this iteration, Signs Circuits was provided during working hours, with tutors given time off from ward duties. Where this was not possible, earlier iterations have scheduled circuits after business hours. Junior doctors are known to feel fulfilled combining their clinical roles with teaching [37], and in the UK, often require evidence of teaching experience to score highly in specialty applications. We have therefore found that sessions were easily staffed in or out of hours.

Variations at other sites have also included teaching larger numbers of patients with clinical signs over a 6-week period or teaching more junior medical students. In addition, other sites have trialled classroom sessions reviewing the previous weeks’ patients, and it should be noted that over a 6-week period with six paired classroom sessions, a large volume of an appropriate medical syllabus can be covered. This is a valuable strategy, where staff are available, facilitating a meaningful cycle of experiential learning and spaced repetition.

Due to the strict infection control issues caused by the COVID-19 pandemic it is unclear in what form PEx teaching will be possible in future. Though infection control must be paramount, the quality of medical students’ teaching has a direct impact on their future competence as doctors and therefore patient safety. This course offers a reproducible way of enhancing medical student examination skills and clinical signs exposure, while limiting the time they are in contact with patients. It has therefore continued intermittently during the academic year 2020–2021.

Lastly, it should be acknowledge that bedside teaching is often criticised for leaving patients in a passive role [38]. However, it also provides an opportunity to role-model how we should respect and value patients and should remain the priority for all involved.

### Limitations

Although this study was underpowered to show a significant change in the number of signs students had been exposed to, it did show a positive trend over what was a small number of sessions. It is also limited by having no control group to evaluate improvements that would have been seen during the students’ placement without this teaching. Further work could evaluate the programme with both larger numbers and a control group.

Further, as with many educational interventions, the use of questionnaires introduces subjective results, and more objective assessment methods, such as examination scores, were not available to us. Some responses will have been limited by recall and are no doubt imperfect reflections of previous teaching and signs exposure. However, if a student cannot remember being exposed to a sign, their ability to recognise it in practice may also be limited, and identification of this as a gap in teaching that needs to be filled is appropriate.

Further, limited numbers of students completed the post-course questionnaire, and the final group were unable to complete the course due to the COVID-19 pandemic, which may have introduced bias.

## Conclusion

There is growing body of literature that PEx skill teaching is declining in medical schools, and the results presented here add weight to this. Developing structures by which PEx teaching can be provided efficiently within the hospital setting is an important step in addressing this. This junior-doctor led high-yield circuit-based structure may help to overcome this challenge by utilising an enthusiastic junior doctor cohort and dedicated leads to provide a volume and quantity of teaching beyond that which could be provided by individual teachers. Otherwise, we risk doctors entering practice able to perform a rote clinical examination, but unable to recognise and interpret the signs they observe within it. We advocate that developing approaches like this is essential to prevent clinical signs becoming myth, and a generation of physicians becoming even more reliant on the computer to treat the patient in front of them.

## Supplementary Material

Supplemental Material
