# Supplementary material for: Will clinical signs become myth? Developing structured Signs Circuits to improve medical students’ exposure to and confidence examining clinical signs
Source: Med Educ Online. 2022 Apr 7;27(1):2050064. doi: 10.1080/10872981.2022.2050064 (PMC9004494; doi:10.1080/10872981.2022.2050064)
Supplement: Supplemental Material [file ZMEO_A_2050064_SM4237.docx]

**Supplemental material:**

**Appendix 1: Survey Questions**

**Pre-course survey**

*Strongly disagree – strongly agree*

- I feel very comfortable examining patients

- I feel very comfortable consistently eliciting clinical signs

- Clinical signs are a pivotal part of diagnosis

- I have received a good amount of teaching on eliciting clinical signs before

- Bedside teaching I have received before has been:

- Of excellent quality
- Suitably interactive
- At an appropriate level

- I feel ready for my final practical OSCEs

- Signs Circuits will be an effective way of delivering clinical examination teaching

What things have made previous bedside teaching good?

What things have made previous bedside teaching you have received bad?

What is the role of clinical signs in evaluating and managing patients?

From your time shadowing in medical school, what importance do you think is placed on the clinical examination?

Do other doctors ever take the time out to show you clinical signs?

What kind of patients / signs do you feel are most useful for you to see?

What kind of patients / signs do you feel are least useful for you to see?

Signs table (see Appendix 2)

**Post-course survey**

*Strongly disagree – strongly agree*

- Number of sessions attended

- I feel very comfortable examining patients

- I feel very comfortable consistently eliciting clinical signs

- Clinical signs are a pivotal part of diagnosis

- The teaching I received was:

- Of excellent quality
- Suitably interactive
- At an appropriate level

- I learned a lot from this course

- I feel ready for my final practical OSCEs

- Signs Circuits will be an effective way of delivering clinical examination teaching

What are your thoughts on the course?

What things made this type of bedside teaching good?

What things could be improved?

What impact will this course have on your own future practice?

What is the importance of the clinical exam in patient management?

What kind of patients / signs were the most useful for you to see?

What kind of patients / signs would you like to see more of?

What kind of patients / signs would you like to see less of?

Signs table (see Appendix 2)

**Appendix 2: Pre-course questionnaire responses to the question: ‘Have you seen the following signs presentations before?’**

| **Signs** | **Number of students reporting prior exposure to sign/condition (n=61)** | **Percent of total (%)** |
| --- | --- | --- |
| **Cardiology** | | |
| Splinter haemorrhages | 22 | 35 |
| Raised JVP | 37 | 59 |
| C-V waves | 1 | 2 |
| Water hammer sign | 7 | 11 |
| Parasternal heave | 9 | 14 |
| Aortic stenosis murmur | 51 | 81 |
| Aortic regurgitation murmur | 27 | 43 |
| Mitral stenosis murmur | 20 | 32 |
| Mitral regurgitation murmur | 36 | 57 |
| Tricuspid regurgitation murmur | 8 | 13 |
| S3 heart sound | 10 | 16 |
| S4 heart sound | 6 | 10 |
| Pericardial friction rub | 8 | 13 |
| Metallic heart valve | 38 | 60 |
| Varicose veins | 41 | 65 |
| Lipodermatosclerosis | 7 | 11 |
| **Abdominal** | | |
| Scleric icterus | 15 | 24 |
| Spider naevi | 32 | 51 |
| Gynaecomastia | 31 | 49 |
| Hepatomegaly | 31 | 49 |
| Splenomegaly | 17 | 27 |
| Shifting dullness | 31 | 49 |
| Fluid thrill | 6 | 10 |
| Polycystic kidneys | 6 | 10 |
| Palpable AAA | 9 | 14 |
| Hernias | 37 | 59 |
| Abdominal scars | 43 | 68 |
| **Respiratory** | | |
| Dahl's sign | 3 | 5 |
| Deviated trachea | 4 | 6 |
| Raised/decreased tactile vocal fremitus | 14 | 22 |
| Raised/decreased vocal resonance | 14 | 22 |
| Bronchial breathing | 32 | 51 |
| Fine inspiratory crepitations | 41 | 65 |
| Coarse inspiratory crepitations | 43 | 68 |
| Whispering pectoriloquy | 2 | 3 |
| **Neurology** | | |
| Increased tone | 49 | 78 |
| Decreased tone | 35 | 56 |
| Lead-pipe rigidity | 24 | 38 |
| Clasp knife rigidity | 15 | 24 |
| Hyper-reflexia | 33 | 52 |
| Hypo-reflexia | 32 | 51 |
| Hoffman's sign | 5 | 8 |
| Positive finger-nose test | 16 | 25 |
| Dysdiadokokinesia | 20 | 32 |
| Babinski response | 17 | 27 |
| Glabellar tap | 6 | 10 |
| Internuclear ophthalmoplegia | 11 | 18 |
| Nystagmus | 30 | 48 |
| Oculomotor palsy | 13 | 21 |
| Trochlear palsy | 5 | 8 |
| Abducens palsy | 6 | 10 |
| Facial nerve palsy | 16 | 25 |
| Ptosis | 29 | 46 |
| Horner's syndrome | 10 | 16 |
| **Other** | | |
| Clubbing | 47 | 75 |
| Koilonychia | 23 | 37 |
| Leukonychia | 19 | 30 |
| Beau's lines | 1 | 2 |
| Lindsay's nails | 0 | 0.0 |
| Terry's nails | 1 | 2 |
| Peripheral oedema | 31 | 49 |
| Lymphoedema | 14 | 22 |
